# Clonal Hypereosinophilia with ETV6 Rearrangement Evolving to T-Cell Lymphoblastic Lymphoma: A Case Report and Review of the Literature

**DOI:** 10.1155/2013/652745

**Published:** 2013-04-21

**Authors:** Filipa Moita, Isabel Bogalho, Helena Alaiz, Joana Parreira, Maria Jesus Frade, Albertina Nunes, Maria Gomes da Silva

**Affiliations:** Hematology Department, Instituto Português de Oncologia de Lisboa, Francisco Gentil, Rua Professor Lima Basto, 1099-213 Lisbon, Portugal

## Abstract

Hypereosinophilia, either clonal or reactive, has been described in association with multiple hematological malignancies. We describe a case of a patient presenting with hypereosinophilia that evolved into T-cell lymphoblastic lymphoma. Complete remission was achieved with chemotherapy; however, hypereosinophilia recurred 5 months later in association with myeloblastic bone marrow infiltration and without evidence of lymphoblastic lymphoma relapse. Cytogenetic analysis of the bone marrow showed a complex translocation involving chromosomes 7, 12, and 16. A rearrangement of ETV6 gene (12p13) was demonstrated by FISH studies, thus confirming the clonality of this population. The association of lymphoblastic lymphoma, eosinophilia, and myeloid hyperplasia has been described in disorders with FGFR1 rearrangements. We hypothesize that other clonal eosinophilic disorders lacking this rearrangement could behave in a similar fashion through different pathogenic mechanisms.

## 1. Background

Hypereosinophilia (HE) can be associated with a wide range of both reactive and malignant disorders. In hematologic malignancies, HE may appear in disorders where the eosinophils are a part of the malignant clone, such as the myeloproliferative neoplasms, or result from stimulation by growth factors or cytokines produced by the malignant clone. These include lymphoproliferative neoplasms, particularly T-cell non-Hodgkin lymphoma, and Hodgkin lymphoma. Eosinophilia has also been described in association with acute lymphoblastic leukemia, more frequently of B-cell origin [1, 2]. Its appearance sometimes precedes the diagnosis of malignancy by several years [3], and thus, after the exclusion of reactive causes, the presence of eosinophilia should lead to the investigation of an underlying clonal disease.

## 2. Case Report

We report the case of a 58-year-old female, with a known history of multiple sclerosis that was at the time asymptomatic and under no specific therapy. She was otherwise healthy, not taking any medication. Her family history was unremarkable. The patient was referred to the hematology department with the diagnosis of T-cell lymphoblastic lymphoma. She had been observed in her local hospital two months earlier, after the incidental discovery of leukocytosis with eosinophilia, bicytopenia, and elevated lactate dehydrogenase (LDH) (Table 1). At the time, a bone marrow aspirate was performed and revealed a hypercellular bone marrow (BM) with marked hypereosinophilia, no increase in the number of blasts, and no specific morphologic alterations. Immunophenotyping and cytogenetic studies were not conducted at this point. The patient was kept under observation and remained asymptomatic, with spontaneous normalization of the hematological parameters and LDH. Two months later a cervical enlarged lymph node appeared. The excisional biopsy showed lymphoblastic lymphoma of T-cell lineage, and she was referred to our hospital for further management.

At admission, the patient presented with fatigue but was otherwise asymptomatic. Specifically, she denied respiratory, gastrointestinal, or other symptoms suggestive of a reactive eosinophilia. She was not a smoker and had no history of allergies. Physical examination revealed enlarged bilateral cervical, supraclavicular, and axillary lymph nodes and an enlarged spleen.

Laboratory abnormalities included anemia, a slight leukocytosis with eosinophilia, and a normal LDH (Table 1). 

The cervical lymph node biopsy was reviewed, and the diagnosis of T-cell lymphoblastic lymphoma was confirmed. A fine needle aspirate was conducted and flow cytometry studies showed infiltration by 92% aberrant immature T-cells that were Tdt/CD99/cCD3/CD5/CD2/CD1a/CD4 positive. The fluorescent in situ hybridization (FISH) analysis for BCR-ABL gene fusion in the lymph node aspirate was negative.

The BM aspirate revealed a normocellular BM with 45.8% eosinophils, relative granulocytic hyperplasia, and no lymphoblastic or myeloblastic infiltration. No dysplastic changes were found. Flow cytometric analysis did not identify an aberrant lymphoid or myeloid population; 0.3% CD34+ precursors were present. BM biopsy findings were interpreted as reactive. 

The investigation of PDGFRA rearrangements by FISH and polymerase chain reaction (PCR) in the bone marrow was negative at this time point. Cytogenetic studies showed a normal karyotype in the 20 metaphases analyzed. 

As part of the lymphoma staging procedures, a computed tomography (CT) scan was performed showing multiple enlarged lymph nodes (cervical, supraclavicular, axillary, mediastinal, abdominal, iliopelvic, and inguinal) as well as splenomegaly. The PET scan revealed some adenopathies with an increased metabolism. Neoplastic cells were not found in the spinal tap.

Treatment was started according to the CALGB 9111 protocol for lymphoblastic lymphoma/leukemia [4], with complete remission documented after induction. The patient proceeded with the early intensifications, according to schedule, without significant toxicities, except for an allergic reaction to the *E.Coli* asparaginase requiring a switch to Erwinase. Central nervous system prophylaxis was performed with high dose methotrexate (MTX), and radiotherapy was not administered due to the known history of multiple sclerosis.

Sixteen days after MTX administration, the patient was admitted with fever without an obvious infectious cause. A progressive increase in the leukocytosis, with neutrophilia, eosinophilia (Table 1), monocytosis, and the appearance of immature myeloid precursors in the peripheral blood (myeloblasts 8%, promyelocytes 3%, myelocytes 3%, and metamyelocytes 4%) followed, as well as a raise in LDH levels. This picture resembled the alterations that occurred two months prior to diagnosis.

**Figure 1:**
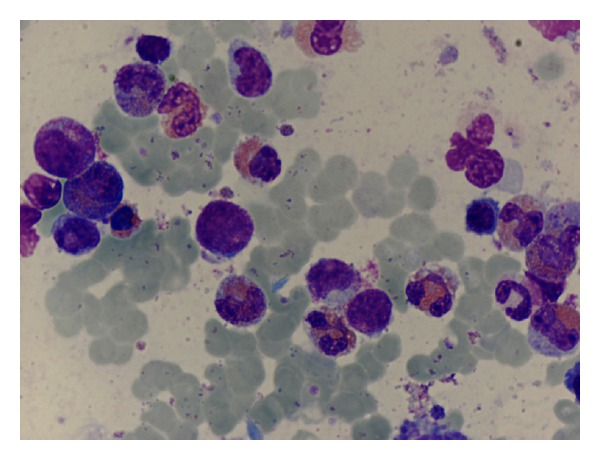
Bone marrow aspirate at relapse showing infiltration by myeloblasts, left shifted granulopoiesis, and marked hypereosinophilia.

**Figure 2:**
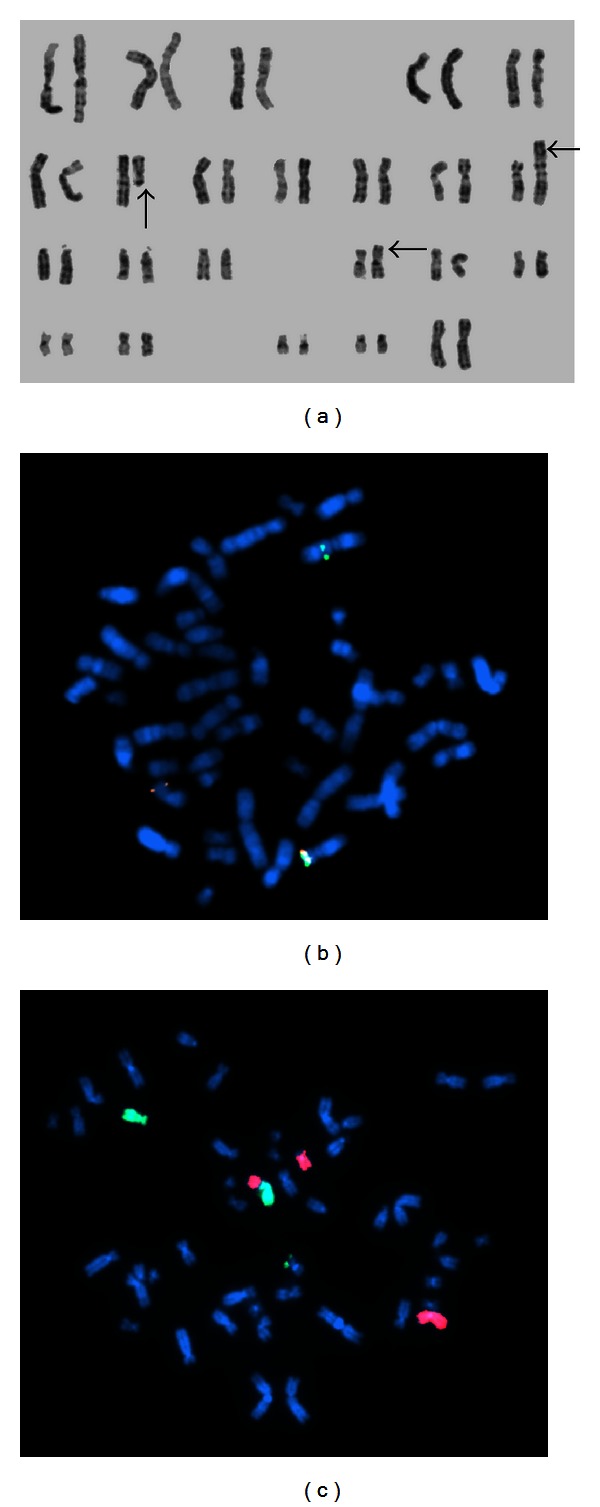
Cytogenetics of the patient's bone marrow (a) G-banded karyotype showing 46,XX,t(7;12)(q22;p13),add(16)(p13); (b) fluorescence in situ hybridization using a dual-color break-apart probe showing rearrangement of ETV6 (12p13) (c) whole chromosome painting of chromosomes 7 (red) and 12 (green) showing 46,XX, del(7)(q22),der(12)t(7;12)(q22;p13)t(12;16)(p13;p13);der(16)t(12;16).

At this time, a repeated BM aspirate showed a hypercellular marrow, with myeloid hyperplasia, and infiltration by 11% of myeloblasts (Figure 1). This finding was confirmed by flow cytometry that identified 10% myeloblasts expressing CD34, CD117, HLA-DR, CD33, and CD13. Prominent eosinophilia was also present, and phenotypic aberrations in the granulocytic maturation were evident. There was no evidence of lymphoblastic infiltration either in morphology or in flow cytometry. Conventional cytogenetic analysis showed 10 metaphases with t(7;12)(q22;p13) as well as the addition of unknown genetic material to the short arm of chromosome 16 (Figure 2(a)). FISH studies using a dual color break-apart probe were positive for the ETV6 (12p13) rearrangement (Figure 2(b)). To further characterize these alterations, whole chromosome painting of chromosomes 7 and 12 was conducted (Figure 2(c)) and revealed a complex pattern of rearrangements involving chromosomes 7, 12, and 16: 46,XX,del(7)(q22),der(12)t(7;12)(q22;p13)t(12;16)(p13;p13),der(16)t(12;16).

The BM samples were also retested for PDGFRA, PDGFRB, and BCR-ABL rearrangements by FISH, with repeated negative results.

A PET scan was repeated at this time point and showed only splenic caption (SUV 4.55). An abdominal ultrasound showed a heterogeneous splenomegaly without nodular lesions, suggesting a spleen infiltrative process.

Despite the introduction of high dose of steroid therapy, there was a progressive increase of leukocytosis with 19% circulating myeloblasts, thrombocytopenia, and anemia, possibly reflecting progression to acute myeloid leukemia, although this finding was not confirmed by bone marrow evaluation. Cytoreduction with hydroxyurea was attempted with a slow but steady decrease in the leukocyte counts.

Eight days later, the patient developed sudden dyspnea with severe respiratory failure. Bilateral pulmonary infiltrates present on chest X-ray and high resolution pulmonary CT scan raised the hypothesis of an infectious process. In spite of antimicrobial therapy, the patient progressed to acute respiratory distress syndrome requiring invasive mechanical ventilation and eventually died in the ICU, about 4 weeks after the febrile episode and 6 months after the diagnosis of lymphoblastic lymphoma. Autopsy was not conducted, so the exact cause of death (infection/disease progression) was not determined.

## 3. Discussion

The association between acute lymphoblastic leukemia (ALL) and eosinophilia was first described by Spitzer and Garson in 1973 [2]. The most common cytogenetic abnormality associated with this presentation is t(5;14)(q31;q32) resulting in an overproduction of IL-3; this entity has been recently recognized as a distinct subtype among B-cell ALL in the 2008 WHO classification [1]. However, t(5;14)(q31;q32) translocation is only present in about 10% of cases of ALL with eosinophilia [5], and several case reports with the same clinical phenotype but lacking the rearrangement have been described [6–9]. The association between lymphoblastic lymphoma (LBL) and eosinophilia appears to be less common. However, the concomitant presence of T-LBL, eosinophilia, and myeloid hyperplasia is known to occur in patients carrying the t(8;13)(p11;q11) translocation [10]. This distinct pathological entity is characterized by the rearrangement of the fibroblast growth factor receptor 1 (FGFR1) gene located at chromosome 8p11 [11].

Other rearrangements may be associated with eosinophilia. Recent reports of ALL with this presentation have implicated chromosomes 7 and 12, namely-7, add(12)(p13) [7] and del(7)(q22) [8]. Recently Bhatti et al. [6] described a patient with B-cell lymphoblastic leukemia/lymphoma and severe eosinophilia and splenomegaly carrying a chromosomal translocation t(7;12)(q22;p13). The karyotype analysis in the current case demonstrated a similar rearrangement. Additionally, the presence of a rearrangement involving the ETV6 gene was demonstrated by FISH.

The ETV6 gene, located at 12p13, belongs to a large family of transcription factors and has been previously implicated in the pathogenesis of multiple hematological malignancies [12], occasionally related with eosinophilia [13]. 

Many translocation partners to ETV6 have been characterized at the molecular level, with the identification of 30 ETV6 partner genes [12]. In our case, we were unable to identify the translocation partner to ETV6.

The association of eosinophilia with immature lymphoid neoplasms could be explained by the presence of an underlying clonal disease affecting a stem cell or even a more mature myeloid precursor that could transform into acute lymphoblastic leukemia/lymphoma [7]. This behavior is similar to what is seen in chronic myeloid leukemia blast crisis or in leukemias/lymphomas associated with FGFR1 rearrangement [1, 10, 11]. We favor this explanation in our patient, in whom the presence of eosinophilia was not concomitant with the lymphoblastic lymphoma and who had a clonal cytogenetic alteration only detected at the time of the bone marrow infiltration by immature myeloid cells.

The absence of chromosomal rearrangements in the initial bone marrow analysis could be due to a putative small size of the malignant clone but may also indicate that the clinical course of our patient was part of the clonal evolution of a chronic disorder, with the appearance of “de novo” cytogenetic alterations in the acute phase. This is in agreement with findings in recently published series, where transformation into acute leukemia was described as a frequent event [13] in patients with the diagnosis of chronic eosinophilic leukemia. Moreover, chromosome 7 abnormalities, particularly at 7q22, have been commonly associated with myelodysplastic syndromes and acute myeloid leukemia [14].

Our patient was treated according to the CALGB protocol, which includes drugs that are active in myeloid malignancies such as daunorubicin in the induction phase and cytarabin in the intensification. We could hypothesize that the proliferation of myeloid clone occurred after these drugs were no longer administered.

In conclusion, our finding of an association between a myeloid clonal proliferation with t(7;12)(q22;p13), ETV6 rearrangement, and eosinophilia in a patient previously diagnosed with LBL is in agreement with the findings of other authors [6–8] and supports the diagnosis of a hematopoietic neoplasm originating in very immature cells with the ability to differentiate in myeloid and lymphoid lineages at different moments. We could hypothesize that this entity behaves in a similar way as the disorders with FGFR1 rearrangements and myeloid hyperplasia, eosinophilia, and lymphoblastic lymphoma, but further studies should be pursued aiming to identify other genes involved in this translocation and the underlying mechanism that leads to eosinophilia.

## Figures and Tables

**Table 1:** Laboratory data.

	December 2011 (local hospital)	February 2012 (diagnosis of ALL)	March 2012 (after induction)	4th July 2012 (D16 SNC prophylaxis)	12th July 2012
WBC (/mm^3^)	97.700	11.350	3.650	37.900 (8% myeloblasts)	159.000 (19% myeloblasts)
Neutrophils (/mm^3^)	11.700	4200	2.850	22.360	44.520
Eosinophils (/mm^3^)	41.000	4540	0	4930	57.240
Hb (g/dL)	8.9	8.0	8.6	12.2	10.5
Platelets (/mm^3^)	118.000	251.000	294.000	96.000	13.000
LDH (UI/L)	2624	229	113	2017	3659

WBC: white blood cell; Hb: hemoglobin; LDH: lactate dehydrogenase.
